# The relationship between coping and distress among faculty members and students during COVID-19 pandemic lockdown: The moderating effect of gender

**DOI:** 10.3389/fpsyt.2023.1103049

**Published:** 2023-02-22

**Authors:** Abdalla A. R. M. Hamid, Abdullah Seif Abdullah Al Miskry, Abdel Hameed M. Darweesh

**Affiliations:** ^1^Department of Clinical Psychology, United Arab Emirates University, Al Ain, United Arab Emirates; ^2^Department of Cognitive Sciences, United Arab Emirates University, Al Ain, United Arab Emirates

**Keywords:** psychological stress, dealing with stress, COVID-19, sex, moderation

## Abstract

**Objectives:**

Previous research has documented significant associations between the COVID-19 pandemic lockdown, various mental health problems, and coping strategies. However, literature on the moderating role of gender on the relationship between distress and coping strategies during COVID-19 is almost nonexistent. Hence, the main objective of this study was two folds. To examine gender differences in distress and coping strategies, and to test the moderating effect of gender on the relationship between distress and coping among university faculty members and students during the COVID-19 pandemic.

**Method:**

A cross-sectional web-based study design was used to collect data from the participants. A sample of 649 participants (68.9% university students and 31.1% faculty members) was selected. The General Health Questionnaire (GHQ-12) and the Coping Inventory for Stressful Situations (CISS) were used to collect data from the participants. The survey was sent out during the COVID-19 lockdown from May 12th to June 30th, 2020.

**Results:**

The results showed significant gender differences in distress and the three coping strategies. Women consistently scored higher on distress (*p* < 0.01), task-focused (*p* < 0.05), emotion-focused (*p* < 0.001), and avoidance coping (*p* < 0.01) compared to men. Gender moderated the relationship between emotion-focused coping and distress (*p* < 0.001) but not the relationship between distress and task-focused or avoidance coping.

**Conclusion:**

Increased emotion-focused coping is associated with decreased distress among women while the use of emotion-focused coping by men predicted more distress. Workshops and programs focused on providing skills and techniques on how to cope with stressful situations induced by the COVID-19 pandemic are recommended.

## 1. Introduction

Currently, the COVID-19 pandemic represents one of the worst challenges to human health worldwide. It is considered the sixth global public health problem (1). The COVID-19 pandemic has been a possible source of psychological stress for humans due to the demands it poses on them that may result in poor mental health (2, 3). Stress is defined as a person-environment relationship that is perceived by the person as demanding and threatening to his/her well-being (4). The positive aspect of this relationship is referred to as eustress (5). Increased distress is found to be associated with the COVID-19 pandemic lockdown (6–9). In fact, evidence from previous research has shown that the vast majority of participants suffered from psychological distress during the COVID-19 lockdown (10, 11). In addition, severe negative mental health consequences were reported in the Italian general population during the COVID-19 lockdown measures (12). Moreover, gender was identified as one of the significant factors in susceptibility to distress and other psychological problems (7).

Literature on gender and distress shows substantial differences between male and female (13–16). For example, during the current COVID-19 pandemic, women reported significantly elevated levels of stress and psychological problems, such as depression and anxiety (2, 3, 17, 18). Research has also revealed significant gender differences in relation to distress in various settings. For instance, psychological distress is more associated with female compared to male university students (19–22). Consistent with this trend, other studies found that gender differences in perceived psychological health, and academic stress are non-significant, though female students reported slightly more stress (19, 23). Likewise, more studies reported increased distress among women in the general population (24, 25).

Stressful events such as the COVID-19 pandemic can trigger mental health problems (26) that necessitate the use of coping resources to tackle such stressful demands. Coping refers to the process of managing the relationship between a person and the environment that is appraised as taxing (4). Research has found significant gender differences in coping (27–31). Women use more emotion-focused coping and social support seeking while men use more problem-focused coping (32, 33). Women also employ avoidance coping more often than men (34). For instance, women in Saudi Arabia reported more use of religious coping and social support compared to men (21). Females also used more problem-focused, emotion-focused strategies (22, 35), and avoidance coping (30). Further, male participants used maladaptive coping strategies, while women used more adaptive coping (22, 35, 36). During the COVID-19 pandemic, females also used more adaptive coping compared to men (6).

Regarding the relationship between coping strategies and distress, research findings indicate that coping strategies were significantly related to psychological distress (37–40). For instance, avoidance and Problem-focused coping strategies were found to relate to psychological distress (38). In contrast, some studies found no relationship between coping strategies and distress (41, 42).

Research has also demonstrated the ability of gender to moderate the relationship between coping and anxiety (43). Along the same line, scholars found that gender moderates the effect of coping on psychological distress among Spanish employees (44). The authors reported that social support had a more beneficial influence on Spanish women than on men. In addition, gender was reported to significantly moderate the relationship between coping and distress (45). Moreover, research has reported a significant gender moderation effect on the relationship between coping and stress. It was indicated that the use of adaptive coping was associated with decreased distress among men (46).

The main purpose of the present study is to examine whether gender moderates the relationship between coping and distress among participants during the COVID-19 lockdown. In addition, we aimed to investigate gender differences in psychological distress and coping strategies. Although the relationship between coping strategies and distress and the COVID-19 pandemic is documented (37–40), the literature on the moderating role of gender on the relationship between distress and coping strategies during COVID-19 is almost nonexistent. Hence, this study may contribute to the understanding of gender roles in coping with mental health problems associated with the COVID-19 pandemic. Based on the literature review the following hypotheses were formed: (1) Significant gender differences are expected in psychological distress and coping amid the COVID-19 pandemic lockdown, (2) coping strategies significantly correlate with distress among participants, and (3) gender moderates the relationship between coping and distress during the COVID-19 lockdown.

## 2. Methods

### 2.1. Study design and participants

A cross-sectional web-based study design was used to collect data from the participants. The sample included 649 participants; 68.9% (n = 447) were university students and 31.1% (202) were faculty members. A convenient sampling method was used to select the participants from different universities in the emirate of Abu Dhabi, UAE. Inclusion criteria were participants aged ≥17 years, being an enrolled university student or a permanent faculty member, and living in the United Arab Emirates during the COVID -19 pandemic lockdown. Exclusion criteria were age < 17 or being not an active student (registration status) or a permanent faculty member, and living outside the United Arab Emirates during the COVID -19 pandemic lockdown. About 17.6% (*n* = 114) of the participants’ ages ranged from 17–18; 45.3% (*n* = 294) aged 19–22 years; 6% (*n* = 39) aged 23–29; 1.8% (*n* = 12) aged 30–39; 14.2% (*n* = 92) aged 40–49, while 15.1% (*n* = 98) aged 50 years or more. About 73.2% (*n* = 475) females and 26.8% (*n* = 174) males took part in this study. Of these participants, 31.1% (*n* = 202) were married while 68.9% (*n* = 447) were single.

### 2.2. Measures

#### 2.2.1. Demographic information

Participants were asked to provide demographic information, namely; status (enrolled student/faculty member), gender, age, and marital status. Next, they responded to the Coping Inventory for Stressful Situations (CISS) and the General Health Questionnaire (GHQ-12).

#### 2.2.2. The coping inventory for stressful situations

The CISS encompasses 48 items rated on a 5-point Likert-type scale (1, not at all; 5: very much) (47). The questionnaire measures three dimensions: task-focused coping (e.g., “Schedule my time better”), emotion-focused coping (e.g., “Blame myself for procrastinating”), and avoidance coping (e.g., “Try to go to sleep”). Each dimension consists of 16 items. Some previous studies used the Arabic version of this scale and reported reliabilities of 0.85 (48) and 0.74 (49). The Cronbach Alpha in the present study is 0.86 (*M* = 158.66; SD = 21.80). The Cronbach Alpha values for task-focused coping, emotion-focused coping and avoidance coping were 0.86, 0.84, and 0.82, respectively.

#### 2.2.3. The general health questionnaire (GHQ-12)

The General Health Questionnaire consists of 12 items used to measure general distress (50). In this study, the Likert-type scaling method (0, 1, 2, 3), which is used in survey research (51) was used to rate the responses. In an Arab sample, Cronbach’s alpha reliability was reported to be 0.94 (52). In the present research, the alpha reliability is 0.86 (*M* = 158.66; SD = 21.80).

### 2.3. Procedure

Upon receiving approval from the university ethics committee, UAEU (Ref No: ERS_2020_6114), the link for the online survey was sent to the faculty members and the students. The survey took place during the COVID-19 lockdown from May 12th to June 30th 2020. Prior to responding to the questionnaires, the participants were required to respond to a consent form. The consent form included a brief description of the nature of the study and explained the voluntary nature of the participation in the study. Further, participants were informed that they were free to withdraw their participation at any stage of the study. Participants were granted confidentiality of the information they provided. No incentives were provided to motivate participants to take part in the study. The study complies with all regulations required to conduct a study on humans. Participants who provided informed consent to take part in the study first responded to a set of questions assessing their socio-demographic characteristics, followed by the General Health Questionnaire (GHQ-12) and the Coping Inventory for Stressful Situations CISS.

### 2.4. Data analysis

Mean, standard deviation, t-test, and Point-biserial correlations among key variables were calculated using (IBM SPSS, version 26.0). The normality of the univariate distribution of the data, kurtosis, and skewness values were calculated, and they were within the normality range (±1.96) (53). To explore the moderating effect of gender on the relationship between coping strategies and distress, the hierarchical regression analyses suggested by Aiken and West (54) were performed. Predictor variables were standardized to reduce multicollinearity, while gender was recoded as a dummy variable (male = 0 and female = 1). Gender was then multiplied by each coping dimension (Task-focused, emotion-focused and avoidance) and interaction terms were generated. For the hierarchical regression equations, the predictor variables (coping strategies) and the moderator variable (gender) were entered in the first step as independent variables. Then, the interaction terms between coping strategies and the moderator variable (gender) were entered in the second step. If the interactions are significant, they will be considered as evidence for gender moderation effects on the relationship between coping strategies and distress (55).

## 3. Results

### 3.1. T-test analysis

T-test analysis was initially conducted to find out gender variations in coping dimensions and psychological distress. Significant differences were evident in psychological distress, avoidance, emotion-focused and task-focused dimensions. Females consistently obtained higher scores on distress and coping strategies compared to males. Cohen’s *d* effect size values ranged from small to medium (see Table 1).

**Table 1:** T-test results of distress and coping differences across gender.

Variables	Gender	*M*	SD	*t*	df	Value of *p*	Cohen’s *d*
Distress	Male	16.40	6.45	−3.313	648	0.001	0.449
	Female	18.82	4.06				
Task	Male	56.29	9.75	−2.033	648	0.042	0.183
	Female	0.58.13	10.38				
Emotion	Male	47.07	10.99	−4.899	648	0.000	0.433
	Female	51.79	10.83				
Avoidance	Male	48.53	12.01	−2.741	648	0.006	0.237
	Female	51.22	10.69				

### 3.2. Correlation analysis

Furthermore, point-biserial correlation was conducted to examine the relationship of gender with coping strategies and distress. Results indicated significant relationships between gender, as a dummy variable, distress, and the three coping dimensions (task-focused, emotion-focused, and avoidance coping). Elevated scores on the above variables were consistently associated with the female gender. It was also found that coping dimensions, as predictor variables, were significantly related to distress (see Table 2). More distress was associated with all three dimensions of coping.

**Table 2:** Point-biserial correlation between coping strategies and gender.

Variables	1	2	3	4	5
1. Gender	–				
2. Distress	0.129^**^	–			
3. Task	0.080^*^	−0.085^*^	–		
4. Emotion	0.189^**^	0.173^**^	0.042	–	
5. Avoidance	0.107^**^	0.252^**^	0.276^**^	0.211^**^	–

*n* = 649, *p < 0.05, **p < 0.01.

### 3.3. Testing the moderating effect of gender

**Figure 1:**
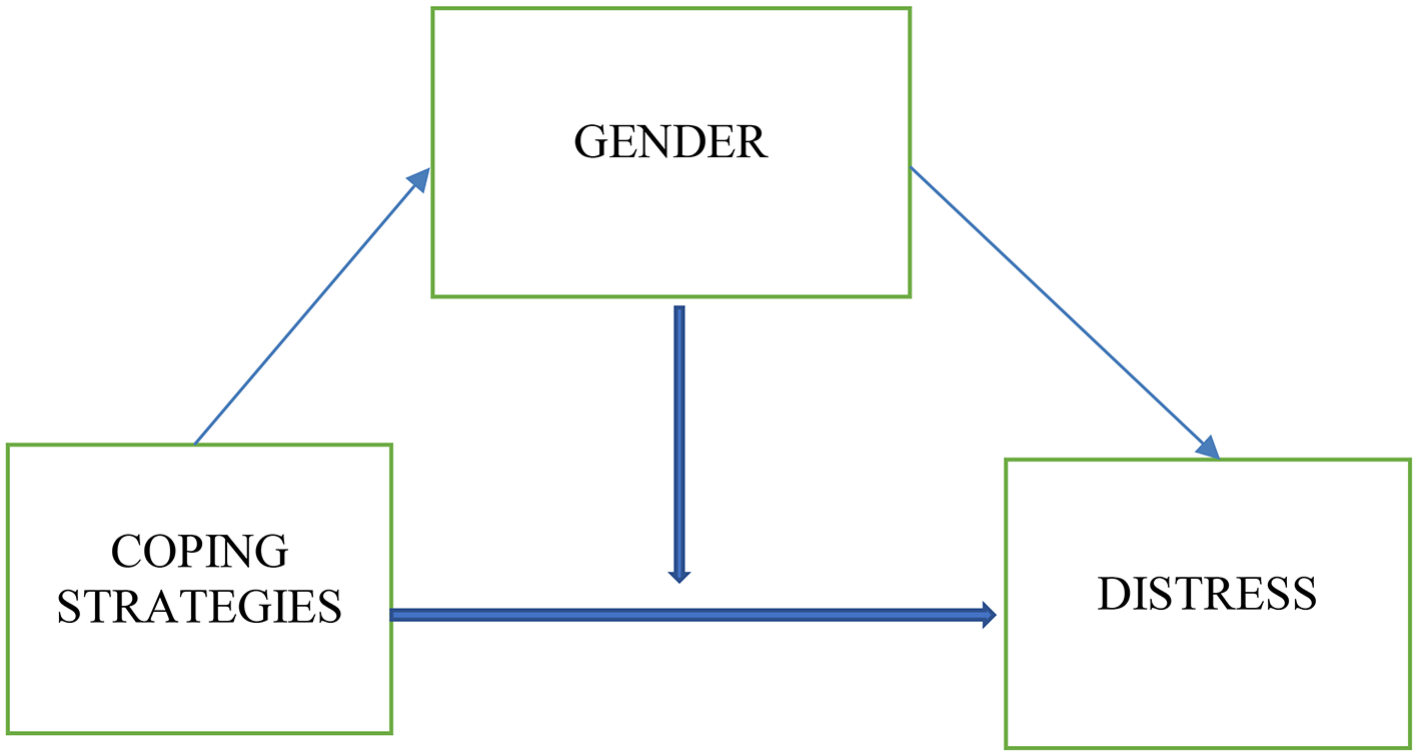
The model of the moderating effect of gender on the relationship between coping and distress.

To explore the moderating effect of gender on the relationship between coping strategies and distress, the hierarchical regression analysis suggested by Aiken and West (54) was performed and the interaction terms were used in the analysis. For the hierarchical regression equations, gender and coping strategies were entered in the first step as independent variables. In the second step, interactional variables between the three coping strategies (Task-focused, emotion-focused and avoidance) and gender were entered. The results (Table 3) showed that gender and all coping strategies predicted distress in the first step. As shown in Table 3, the results indicated a significant increment in the ΔR change in the second step when the interaction terms were included (Δ*R*^2^ = 0.041, Δ*F* = 10.434, *p* < 0.001). Emotion-focused and avoidance coping were still able to predict distress. In addition, the interaction of gender and emotion-focused coping was significant. The interaction value indicated by the t-test was −4.218 which significantly differed from 0. This indicates the existence of the moderating effect of gender in the association between emotion-focused coping and distress. The interaction values indicate that task-focused and avoidance coping were not moderated by gender in their relationship with distress. Figure 1 shows a visual representation of the moderating effect of gender on emotion-focused coping among women.

**Table 3:** The moderating effect of gender on the relationship between coping and distress.

Variables	*β*	*t*	*R* ^2^	Δ*R*^2^	Δ*F*	Value of *p*
Step 1			0.112	0.112	20.339	0.000
Gender- dummy	0.094^*^	2.481				
Task	−0.171^**^	−4.416				
Emotion	0.106^**^	2.745				
Avoidance	0.267^**^	6.742				
Step 2			0.153	0.041	10.434	0.000
Gender-dummy	0.053	1.391				
Task	−0.051	−0.680				
Emotion	0.396^**^	4.704				
Avoidance	0.294^**^	3.775				
Interaction-task	−0.113	−1.500				
Interaction-emotion	−0.335^**^	−4.218				
Interaction-avoidance	−0.083	−1.109				

*n* = 647, *p < 0.05, **p < 0.01.

## 4. Discussion

The main goal of the present research was to explore the moderating effect of gender on the relationship between distress and coping strategies among university faculty members and students during COVID-19 pandemic. In addition, we aimed to determine gender variations in psychological distress and coping strategies used by the participants. The findings of this study contribute to the advancement of the understanding of how gender influences the relationship between psychological distress and coping during the COVID 19 pandemic lockdown.

### 4.1. Gender differences in distress and coping

Prior to examining the moderating effects of coping strategies, gender differences in distress and coping were tested to find out if there is any significant difference between the two groups. The findings indicate significant gender differences in distress. The women’s experience of more distress associated with the COVID-19 pandemic lockdown may suggest that they are more vulnerable to distress than men. These results support the first hypothesis in this study which suggests the existence of significant gender differences in psychological distress and coping amid the COVID-19 pandemic lockdown. The gender differences in distress are congruent with several previous findings (2, 19–21, 56). However, some studies reported a lack of significant gender differences in distress (19, 23).

The findings also indicate significant differences in task-focused, emotion-focused, and avoidance coping pertaining to gender. These results also support the first hypothesis in the present study. The differences between males and females in coping may be due to gender-role differences between men and women. The tendency of female participants to employ a greater degree of emotion-focused coping compared to men is consistent with previous research (22, 56). In addition, other scholars found that women used more problem-focused coping compared to men (6, 36). Regarding avoidance, the findings of the current research are in line with the findings of a previous study where women used more avoidance coping in the face of stress (35). However, some studies found no gender differences in coping (18, 23). The inconsistency in how male and female use coping strategies to deal with distress may be due to contextual (57) and cultural differences between the populations in which those studies were conducted (44). In addition, male and female may learn to manage distress using different coping strategies. They may also experience distress related to the COVID-19 pandemic differently. Further, male and female may experience different levels of awareness of emotional reactions related to the COVID-19 crisis.

### 4.2. Coping strategies and distress among university faculty members and students

The second hypothesis stated that coping strategies significantly relate to distress among participants during the COVID-19 pandemic quarantine. The findings of the correlation and simple regression analyses indicated significant relationships between distress and the three dimensions of coping (task-focused, emotion-focused, and avoidance coping). These results support the second hypothesis and are in line with the findings of many previous studies (37–39, 58, 59). However, the current results did not support the previous findings reported by some authors who found no relationship between coping strategies and distress (41, 42).

### 4.3. Gender moderation of the relationship between coping and distress during the COVID-19 lockdown

The hierarchical regression analysis results suggested a significant increment in the ΔR change in the subsequent step when the interaction terms were included (Δ*R*^2^ = 0.041, Δ*F* = 10.434, *p* < 0.001). The interaction of gender and emotion-focused coping significantly differed from 0. This suggests that gender moderates the relationship between emotion-focused coping and distress. The value of p of the interaction between gender and emotion-focused coping was significantly negative (−0.335, *p* < 0.01). This means an increase in the use of emotion-focused coping is associated with decreased distress among women, while increased use of emotion-focused coping by the male participants may predict increased distress. The pattern of the present results provides evidence for the main effect of gender in moderating the relationship between emotion-focused coping and distress among university faculty members and students. These findings partially support the third hypothesis which postulates that gender moderates the relationship between distress and coping. In general, our results support the findings of previous studies (43–45). Contrarily, the interaction terms indicate that the relationships of distress with task-focused and avoidance coping were not moderated by gender. This lack of moderating effect contradicts the previous findings that reported a significant negative association between adaptive coping and distress among men (46).

### 4.4. Limitations

The study produced very significant findings that contribute to the existing literature. However, it is not devoid of some limitations. This study was conducted during the pandemic lockdown and the only possible means to collect the data was through online surveys. The findings of this study should be considered with caution because of the following limitations:

First, the low response rate associated with the use of online surveys may negatively affect the generalizability of the findings (60, 61). Second, this study is cross-sectional in nature. Therefore, the causal relationships between the study variables (gender, coping strategies, and distress) could not be inferred. More advanced research designs may be suitable for exploring causality. Third, the low magnitude of some effect size coefficients may further limit the ability to generalize some findings.

Finally, the use of convenience sampling in the present study limits the ability to provide the perfect representation of the population. Future studies could use random sampling for better generalizability of the findings such as stratified or cluster random sampling.

### 4.5. Implications

The findings of the study may have several implications. The study suggests that an increase in using emotion-focused coping is associated with decreased distress among women and increased distress among men. Therefore, workshops and programs aimed at providing useful skills and techniques on how to cope with stressful situations caused by the COVID-19 pandemic are required. The findings of the current study also suggest that females experience higher levels of distress than males during the COVID-19 pandemic lockdown. Thus, preventive measures might be useful in building strong inoculation against distress among female participants. In addition, males need to be trained in task-focused coping so that they can use more appropriate coping strategies. Furthermore, the participants’ gender must be considered in designing programs that best meet the needs of each gender. Future studies may be useful to further explore the effects of other factors such as age, marital status, socio-economic status, employment, etc. on the relationship between coping and distress associated with the COVID 19 pandemic.

### 4.6. Conclusion

The findings of this study revealed that gender moderates the relationship between emotion-focused coping and distress. It is evident that increased emotion-focused coping is associated with decreased distress among women while the use of emotion-focused coping by men predicts more distress. This suggests that fostering emotion-focused coping strategies in women may serve as a protective factor against psychological distress during health crises, while emotion-focused coping may be harmful to men. Therefore, men need to be acquainted with more constructive coping skills such as problem-focused coping.

## Data availability statement

The raw data supporting the conclusions of this article will be made available by the authors, without undue reservation.

## Ethics statement

The studies involving human participants were reviewed and approved by The Social Sciences Research Ethics Committee, United Arab Emirates University. The patients/participants provided their written informed consent to participate in this study.

## Author contributions

AH, AM, and AD contributed to the manuscript planning, data collection, conceptualization, data analysis, methodology, writing the original draft and review, and editing. All authors contributed to the article and approved the submitted version.

## Conflict of interest

The authors declare that the research was conducted in the absence of any commercial or financial relationships that could be construed as a potential conflict of interest.

## Publisher’s note

All claims expressed in this article are solely those of the authors and do not necessarily represent those of their affiliated organizations, or those of the publisher, the editors and the reviewers. Any product that may be evaluated in this article, or claim that may be made by its manufacturer, is not guaranteed or endorsed by the publisher.
